# Association Between Neck Circumference, Cardiovascular Risk Factors, and Relative Muscle Strength in Older Women

**DOI:** 10.1155/jare/2875747

**Published:** 2026-04-10

**Authors:** Odilon Abrahin, Naicha Stefanie Félix Souza, Simone Vitória Pantoja Sidônio, Rejane Pequeno Rodrigues Abrahin

**Affiliations:** ^1^ Laboratory of Resistance Exercise and Health (LERES), State University of Pará (UEPA), Belém, Pará, Brazil; ^2^ Graduate Program in Rehabilitation and Functional Performance, State University of Pará (UEPA), Belém, Pará, Brazil; ^3^ Graduate Program in Science and Mathematics Education, Federal University of Pará (UFPA), Belém, Pará, Brazil, ufpa.br

**Keywords:** aging, anthropometry, cardiovascular risk, elderly women, functional capacity, neck circumference

## Abstract

**Background:**

Neck circumference (NC) has been proposed as a potential anthropometric marker of cardiovascular risk, but its relationship with functional capacity in older women remains unclear.

**Objective:**

To analyze the association between NC, cardiovascular indicators, and functional capacity in older women.

**Methods:**

A cross‐sectional observational study was conducted with 64 postmenopausal women aged over 60 years. Participants were stratified into two groups according to NC values < 33.5 cm (*n* = 33) and ≥ 33.5 cm (*n* = 31). Anthropometric, biochemical, and blood pressure assessments were conducted, alongside physical and functional capacity tests, including sit to stand, biceps curl, timed up and go (TUG), six‐minute walk test (6MWT), and relative muscle strength. Comparisons and correlations were analyzed using appropriate statistical tests (*p* ≤ 0.05).

**Results:**

Older women with NC ≥ 33.5 cm presented higher body mass, body mass index, waist circumference, sum of skinfolds, and diastolic blood pressure. Relative muscle strength was lower in this group, with effect sizes suggesting a meaningful difference. No consistent differences were observed between groups in sit to stand, biceps curl, TUG, and 6MWT performance. NC was positively associated with cardiovascular risk markers and inversely associated with relative muscle strength, indicating a consistent pattern of association.

**Conclusion:**

Older women with higher NC values showed greater cardiovascular risk and lower relative muscle strength, but no difference in functional test performance (sit to stand, biceps curl, TUG, and 6MWT performance). These findings support NC as a practical and low‐cost anthropometric indicator of cardiovascular risk in older women.

## 1. Introduction

By 2030, one in six people worldwide is expected to be aged 60 years or older. Moreover, the number of individuals aged 80 years and above is projected to triple between 2020 and 2050, reaching 426 million and surpassing the global population of children for the first time [1]. This demographic transition has significant public health implications, particularly due to the expected increase in chronic conditions such as cardiometabolic diseases [2]. In this context, the development of effective and accessible strategies for risk assessment, prevention, and clinical monitoring in the older population becomes essential.

Aging induces morphofunctional changes, including reduced muscle mass and increased central adiposity, that elevate cardiovascular risk and impair functional capacity. [3]. These changes contribute to increased cardiovascular risk and reduced functional capacity [4]. Routine assessment of these risks is traditionally performed using anthropometric techniques, including waist circumference and neck circumference (NC) [4–6]. Increased NC has been associated with elevated blood pressure, insulin resistance, and metabolic syndrome [4, 5].

Traditionally, waist circumference has been widely used as an indicator of central adiposity and cardiometabolic risk [6]. However, NC has emerged as a complementary [4] and, in some contexts, advantageous anthropometric marker. NC reflects upper‐body subcutaneous fat accumulation, which is metabolically active and strongly associated with cardiometabolic alterations, including insulin resistance and blood pressure dysregulation [4, 5]. In addition, upper‐body fat accumulation assessed by NC has been linked to inflammatory pathways and cardiometabolic dysregulation, reinforcing its relevance as a simple marker of cardiovascular risk in older women. Moreover, NC can be measured quickly, without the need for extensive participant preparation, which enhances its applicability in clinical and epidemiological studies.

Women experience significant physiological changes during the menopausal transition that directly affect adiposity distribution [7]. The decline in estrogen levels leads to greater visceral fat accumulation and distinct metabolic alterations compared to premenopausal women and age‐matched men. These changes are associated with increased adiposity, pro‐inflammatory profiles, and higher cardiometabolic risk [7].

In addition, functional capacity, defined as the ability to perform activities of daily living independently, has emerged as a key clinical marker of health in older adults due to its strong association with quality of life and autonomy [8]. Functional decline including reductions in muscle strength, mobility, and cardiorespiratory endurance is linked to adverse outcomes such as falls, recurrent hospitalizations, and premature mortality [9]. Early detection of these impairments through functional assessments is therefore essential for preventing complications and guiding effective, targeted interventions.

To the best of our knowledge, no study has examined the relationship between NC, cardiovascular risk factors, and functional aspects in older women. Our main hypothesis is that higher NC values are associated with increased cardiovascular risk and reduced functional capacity in older women. Therefore, the aim of our study was to analyze the association between NC, cardiovascular indicators, and functional capacity in older women.

## 2. Materials and Methods

This is a cross‐sectional observational study conducted in accordance with the Strengthening the Reporting of Observational Studies in Epidemiology (STROBE) guidelines (Supporting Table 1). This study was performed at the Laboratory of Resistance Exercise and Health, a primary care center, in the city of Belém, State of Pará, Brazil, including 83 individuals who visited the outpatient clinic for any reason from January 2025 to June 2025. A total of 64 elderly women met the inclusion criteria, completed all assessments and examinations, and were included in our study.

All participants signed an informed consent form. The study protocol was approved by the Ethics Committee of the University (CAAE 30059120.3.0000.5701). Participants were stratified according to NC values using a cutoff point of 33.5 cm. This threshold was originally proposed by Saad et al. [10] in a Brazilian primary care population, where NC ≥ 33.5 cm in women demonstrated good sensitivity and specificity for predicting insulin resistance, a major cardiometabolic risk factor. Based on this classification, our sample was divided into two groups: NC < 33.5 cm (*n* = 33) and NC ≥ 33.5 cm (*n* = 31), for the analysis of physical capacities, functionality, and cardiovascular risk factors.

### 2.1. Participants

Recruitment was conducted with the aim of reaching individuals residing in the city and surrounding areas (Belém, State of Pará, Brazil), which is located in the eastern Amazon region. The strategy included printed invitations and dissemination through social media. Informative printed materials were prepared and distributed in strategic locations, such as around the university and in commercial establishments.

This study may be subject to selection bias, as participants were volunteers who might be more motivated and healthier than the general older adult population of Belém. This bias was mitigated by applying clear inclusion/exclusion criteria and using a broad recruitment strategy across different city areas.

The following inclusion criteria were adopted: (a) age > 60 years, in accordance with the World Health Organization [11] definition for low‐ and middle‐income countries, including Brazil; (b) postmenopausal elderly women, determined by self‐report and clinically defined as the absence of menstrual periods for at least 12 consecutive months, not attributable to pregnancy or lactation, or a history of surgical menopause; (c) no participation in any structured physical activity in the past three months; (d) full ability to respond to questionnaires and research instruments, as well as the capacity to perform physical and functional tests; and (e) completion of all laboratory tests. Exclusion criteria were (a) presence of cardiorespiratory diseases that could limit performance during physical tests; (b) neck deformity, goiter, and/or parotid gland hypertrophy; and (c) failure to complete all evaluations.

The sample consisted exclusively of postmenopausal women, a group that presents specific physiological and hormonal characteristics that directly influence body fat distribution and central and cardiometabolic indicators. The participants had preserved functional capacity, allowing them to live independently. In addition, this approach ensured sample homogeneity and consequently reduced potential biases arising from sex‐related differences in body composition and functional performance.

**FIGURE 1:**
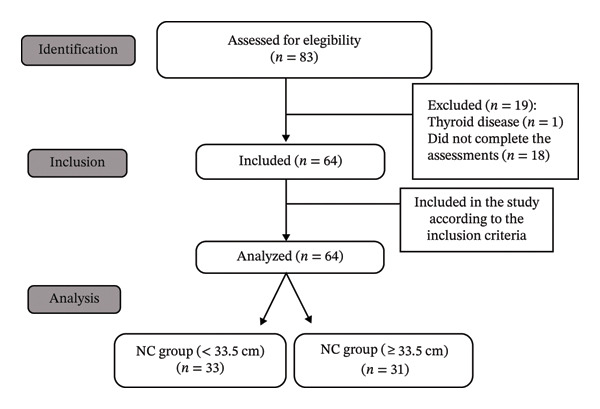
Flowchart of the eligibility process for selecting older women.

Initially, 83 individuals agreed to participate in the study. Of these, 19 participants were excluded: one due to thyroid disease and 18 because they did not complete all stages of the protocol. Among these 18 participants, 10 did not attend the scheduled laboratory examinations, 5 did not complete the anthropometric assessment, and 3 failed to attend the functional testing session. Thus, 64 older adults met the eligibility criteria, completed all evaluations, and were included in the final analysis. Participants were subsequently divided into two groups: NC < 33.5 cm (*n* = 33) and NC ≥ 33.5 cm (*n* = 31) (Figure 1).

### 2.2. Assessments

A battery of tests and examinations was conducted in the following sequence: (1) anamnesis and physical tests; (2) physical retests; (3) blood pressure and anthropometric assessment; and (4) laboratory tests. All procedures were performed with an interval of 48–72 h between sessions. All instruments were calibrated according to the manufacturer’s specifications before data collection.

### 2.3. Sit to Stand in 30 s

First, the older participant sat in the middle of the seat, with a straight spinal column, feet resting on the ground, and arms crossed against the chest. When signaled, the participant was encouraged to fully sit and stand as many times as possible in 30 s [12]. This valuation utilized a 43‐cm‐high chair according to Rikli and Jones. The intraclass test correlation coefficient was 0.86 (IC 95%: 0.76–0.91).

### 2.4. Biceps Curl Test

Individually, the older participant moved one dumbbell (2 kg for women) through a full elbow range of motion during 30 s [12]. The test score constituted the maximal number of full curls with an extended arm position. The intraclass test correlation coefficient was 0.70. (IC 95%: 0.63–0.85).

### 2.5. Timed Up and Go (TUG) Test

The test involved rising from a chair and walking as fast as possible to a cone 3 m away, circling around the cone, and returning to sit on the chair fixed to the ground. Initially, the volunteer stayed in the chair with his/her feet on the floor and their back against the chair [12]. At the beep “go” sign, the older stood up and first moved right and around the cone, back to the chair, and sat down again. This trial was carried out in triplicate, and the mean time was documented. The intraclass test correlation coefficient was 0.96 (IC 95%: 0.95–0.98).

### 2.6. Relative Muscle Strength (RMS)

Handgrip strength was evaluated by a dynamometer (Saehan Corporation, Yeongdeok‐Dong, Korea). Verbal encouragement was used for all participants, and they carried out the test while sitting. The participant squeezed the dynamometer with maximum isometric strength, sustaining a grip for 5 s. This assessment was carried out in triplicate for each hand with 1‐min rest intervals between measurements as used in the previous study [13]. The dynamometer handle was adjusted if required, and the best value was recorded. The intraclass test correlation coefficient was 0.95 (IC 95%: 0.93–0.97).

To obtain RMS, the following formula was used (RMS = absolute strength [kg]/body mass [kg]). RMS adjusted to body size can provide more accurate information for screening sarcopenic obesity [14].

### 2.7. Six‐Minute Walk Test (6MWT)

The participants were instructed to walk as far as they could within a 6‐min period. The evaluation begins with a brief explanation of the test and the importance of walking at a comfortable and sustainable pace throughout the duration [12]. During the execution, an evaluator times the test and encourages the participant to keep walking, providing positive feedback to maintain motivation.

This test assesses cardiovascular endurance by measuring the total distance walked in 6 minutes at a self‐selected pace. After 6 min, the evaluator records the distance walked in meters or other appropriate units. The intraclass test correlation coefficient was 0.73 (IC 95%: 0.62–0.84).

### 2.8. Blood Pressure

Systolic blood pressure and diastolic blood pressure were determined by an automatic blood pressure device (Microlife 3AC1‐1, Widnau, Switzerland) validated by the British Hypertension Society, INMETRO‐ML 275/09, and ANVISA following Brazilian Hypertension Guideline recommendations [15]. Blood pressure measurements were obtained exclusively from the left arm for all participants to ensure methodological standardization and consistency across assessments, in accordance with the manufacturer’s recommendations.

All blood pressure assessments were conducted in the morning (between 08:00 and 10:00 h). Participants were advised to refrain from programmed exercise and caffeine consumption before the measurements [16]. Measurements were obtained twice using a validated automated blood pressure monitor, and the average of the two readings was considered for the analyses [16].

### 2.9. Anthropometry

All measurements were performed by Level 1 and/or Level 2 anthropometrists accredited by the International Society for the Advancement of Kinanthropometry (ISAK), with a technical error of measurement (TEM) ≤ 5% for skinfolds and ≤ 1% for circumferences, ensuring reliability. Participants were instructed to avoid physical activity and alcohol for 24 h prior to testing in order to minimize effects on body temperature and peripheral blood flow [6]. Height and body mass were measured using a precision electronic scale (Líder, São Paulo, Brazil). Neck and waist circumferences were measured using a steel anthropometric tape (Cescorf, Rio Grande do Sul, Brazil). Skinfolds (biceps, triceps, thigh, and calf) were assessed using a clinical caliper (Innovare Cescorf, Rio Grande do Sul, Brazil) and recorded in millimeters. All measurements followed ISAK standardization protocols [6].

### 2.10. Biochemical Parameters

Venous blood samples were collected after a 12‐h overnight fast. A total blood volume of 5–10 mL was obtained via venipuncture and collected into 10‐mL vacutainer tubes containing anticoagulant. The samples were drawn by an experienced technician and stored at −20°C until biochemical analysis, with a maximum storage period of two days. Glycated hemoglobin, fasting glucose, C‐reactive protein (CRP), urea, creatinine, and lipid profile (LDL‐C, HDL‐C, total cholesterol, and triglycerides) were analyzed.

This analysis was performed using an automated analyzer (CMD 600i; Wiener Lab, São Paulo, Brazil) and commercial reagent kits (Wiener Lab, São Paulo, Brazil). The procedures were performed according to the manufacturer’s protocol to ensure the validity of the test results. The analytical sensitivity and detection limits for each biomarker were within the ranges specified by the manufacturer.

Internal quality control procedures were applied daily, and calibration was performed according to the manufacturer’s recommendations. The participating laboratory maintains a rigorous quality control program, with reported average intra‐assay coefficients of variation ranging from 1.2% to 2% and interassay coefficients of variation below 5% for all analytes.

### 2.11. Statistical Analysis

For analytical purposes, RMS was defined a *priori* as the primary outcome of the study. The significance level was set at *p* < 0.05; however, given the exploratory nature of the analyses, emphasis was placed on effect sizes and confidence intervals (CIs) rather than solely on *p* values. Continuous variables were summarized using measures of central tendency and dispersion, with 95% CIs. Data normality was assessed using the Shapiro–Wilk test. The triglyceride–glucose (TyG) index was calculated as Ln (fasting triglycerides [mg/dL] × fasting glucose [mg/dL]/2) [17]. NC was treated as the primary exposure variable. Associations between NC and cardiometabolic, anthropometric, and functional outcomes were examined using correlation analyses for continuous variables and group comparisons based on the predefined cutoff (< 33.5 vs ≥ 33.5 cm).

Categorical variables were presented as absolute and relative frequencies (%). To compare the prevalence of chronic diseases (diabetes, hypertension, and dyslipidemia) between groups (< 33.5 and ≥ 33.5 cm), the chi‐square test or Fisher’s exact test was used, as appropriate. Differences in proportions between groups were calculated with 95% CIs. For the analysis of the number of medications used, frequencies were compared between groups.

For variables that followed a normal distribution, comparisons were performed using the independent samples Student’s *t*‐test, and effect size was calculated using Cohen’s *d*, interpreted as small (0.2), medium (0.5), and large (0.8). For variables with a non‐normal distribution, the Mann–Whitney *U*‐test was used, and the effect size was calculated using the rank‐biserial correlation (*r*), interpreted as small (0.1), medium (0.3), and large (0.5).

In addition, correlations between NC and cardiovascular risk factors, as well as RMS, were assessed using Pearson’s or Spearman’s correlation coefficients, as appropriate. For the Mann–Whitney *U*‐test and Spearman’s correlations, 95% CIs were estimated using bias‐corrected and accelerated (BCa) bootstrapping with 1000 resamples. All variables were complete, with no missing data. Data were analyzed using Jamovi, version 2.4.11, and SPSS, version 20.0 (IBM Corp., Armonk, NY, USA).

Given the evaluation of multiple endpoints, all analyses were considered exploratory. Therefore, no formal correction for multiple comparisons (e.g., false discovery rate) was applied. Instead, the interpretation of results prioritized the magnitude, direction, and precision of the associations, based on effect sizes and 95% CIs, rather than relying solely on statistical significance. No imputation procedures were required because all variables for the participants included in the final analyses were complete.

Sample size was estimated using G ∗ Power (version 3.1) based on a point‐biserial correlation model, assuming a medium effect size (*r* = 0.30), *α* = 0.05, and power (1 − *β*) = 0.80. Based on these parameters, a minimum of 64 participants was estimated.

### 2.12. Bias and Strategies for Minimization

Selection bias may have occurred because recruitment was limited to a single city, which could restrict representativeness; to minimize this risk, all eligible participants were invited and uniformly assessed according to prespecified criteria. Measurement bias was addressed through the use of trained evaluators, validated instruments, and internationally standardized protocols (e.g., ISAK), which increased the accuracy and reliability of the data collection.

Confounding bias could not be completely eliminated as not all external variables were controlled for. Potential unmeasured confounders include smoking status, alcohol consumption, and socioeconomic factors. However, relevant covariates such as age, body mass index, waist circumference, blood pressure, triglycerides, glycated hemoglobin, and fasting glucose were recorded and considered in secondary analyses, which strengthens the robustness of our findings. These strategies enhanced the internal validity of the study, although limitations inherent to the cross‐sectional design remain.

## 3. Results

All participants had a mean age of 69.0 ± 5.9 years (IC 95%: 65–72 years). Baseline clinical characteristics, including comorbidities and medication use according to NC groups, are presented in Supporting Table 2.

Table 1 presents the anthropometric, blood pressure, and biochemical characteristics of the groups with different NC values. No significant differences were observed between the groups in relation to age, systolic blood pressure, glycated hemoglobin, fasting glucose, total cholesterol, triglycerides, CRP, TG/HDL ratio, and index TG/glucose.

**TABLE 1:** Sample characteristics according to neck circumference values.

Variables	Neck circumference				*p*	Effect size *d/r*
	< 33.5 (*n* = 33)	IC 95%	≥ 33.5 (*n* = 31)	IC 95%		
Years (old)	69.8 ± 6.6	67.4–72.1	68.1 ± 5.0	66.2–69.9	0.25	0.29^a^
Body mass (kg)	55.1 ± 6.9	52.8–57.6	66.4 ± 6.3	64.1–68.5	< 0.05^∗^	0.65^b^
BMI (kg/m^2^)	24.3 ± 3.5	22.9–25.5	28.5 ± 2.6	27.6–29.4	< 0.05^∗^	0.56^b^
Neck circumference (cm)	31.7 ± 1.5	31.1–32.2	35.2 ± 1.3	34.7–35.6	< 0.05^∗^	0.86^b^
Waist circumference (cm)	79.5 ± 7.7	76.7–82.2	89.4 ± 7.4	86.5–92.3	< 0.05^∗^	−1.27^a^
∑ Skinfolds (mm)	63.0 ± 18.2	56.5–69.5	83.2 ± 15.9	77.3–89	< 0.05^∗^	−1.1^a^
SBP (mmHg)	122 ± 16.2	116–128	130 ± 15.4	124–135	0.06	−0.48^a^
DPB (mmHg)	72.4 ± 7.0	69.8–75	77.7 ± 9.9	74.1–81.4	< 0.05^∗^	−0.62^a^
Glycated hemoglobin (%)	6.2 ± 0.9	5.9–6.5	6.6 ± 1.3	6.2–7.1	0.13	0.19^b^
Fasting glucose (mg/dL)	101.8 ± 30.1	91.4–112.4	108.7 ± 36.9	98.0–121.5	0.11	0.20^b^
Total cholesterol (mg/dL)	184 ± 30	173–195	171.0 ± 37.4	157–185	0.13	0.39^a^
Triglycerides (mg/dL)	103.4 ± 41.0	90.0–118.3	123.6 ± 48.6	108.1–139.2	0.06	0.24^b^
CRP (mg/L)	3.8 ± 8.5	1.2–7.0	2.7 ± 3.8	1.4–4.1	0.30	0.12^b^
Ratio TG/HDL	1.9 ± 1.0	1.6–2.2	2.3 ± 1.0	1.9–2.7	0.09	0.21^b^
Index TG/glycemia	8.4 ± 0.5	8.2–8.6	8.7 ± 0.5	8.4–8.9	0.12	−0.39^a^

*Note:* Legend: ∑ Skinfolds = sum of skinfolds (triceps, subscapular, abdomen, and thigh); TG/HDL = triglycerides/high‐density lipoprotein. Non‐normal variables, 95% CIs estimated by bias‐corrected accelerated (BCa) bootstrapping, 1000 samples.

Abbreviations: BMI, body mass index; CRP, C‐reactive protein; DBP, diastolic blood pressure; SBP, systolic blood pressure.

^a^Cohen’s *d*.

^b^Mann–Whitney *r*.

^∗^ *p* < 0.05 = statistically significant.

RMS was lower in older women with higher NC values (Table 2), with effect sizes ranging from small to moderate (Cohen’s *d* = 0.34–0.63), suggesting a meaningful difference between groups. No consistent differences were observed for the other physical and functional tests, including the sit to stand, biceps curl, 6MWT, absolute handgrip strength, and TUG, with effect sizes generally small.

**TABLE 2:** Functional physical characteristics according to neck circumference values.

Variables	Neck circumference				*p*	Effect size
	< 33.5 cm (*n* = 33)	IC 95%	≥ 33.5 cm (*n* = 31)	IC 95%		*d/r*
Sit to stand (rep)	17.3 ± 4.8	15.6–19	16.7 ± 4.6	14.9–18.4	0.59	0.13^a^
Biceps curl (rep)	19.3 ± 4.1	17.9–20.8	21.3 ± 3.4	20–22.5	0.07	−0.44^a^
6MWT (m)	485 ± 53.2	466–504	484.0 ± 64.7	460–508	0.96	0.01^a^
TUG (s)	6.8 ± 0.9	6.5–7.2	7.0 ± 1.2	6.6–7.4	0.90	0.02^b^
Handgrip right (kg)	22.2 ± 4.3	20.6–23.7	23.5 ± 3.8	22.1–25	0.19	−0.32^a^
Handgrip left (kg)	20.1 ± 4.1	18.7–21.6	21.5 ± 5.1	19.5–23.4	0.25	−0.28^a^
RMS (left side)	0.37 ± 0.08	0.34–0.40	0.31 ± 0.09	0.27–0.35	< 0.05^∗^	0.63^a^
RMS (right side)	0.40 ± 0.09	0.37–0.44	0.33 ± 0.07	0.30–0.36	< 0.05^∗^	0.34^b^

*Note:* Legend: 6MWT = six‐minute walk test; TUG = timed up and go. Non‐normal variables, 95% CIs estimated by bias‐corrected accelerated (BCa) bootstrapping, 1000 samples.

Abbreviation: RMS, relative muscle strength.

^a^Cohen’s *d*.

^b^Mann–Whitney *r*.

^∗^ *p* < 0.05 = statistically significant.

In the analysis of associations between NC and cardiovascular risk indicators (Table 3), NC showed positive associations with body mass, body mass index (BMI), waist circumference, sum of skinfolds, systolic and diastolic blood pressure, and glycated hemoglobin. An inverse association was observed with RMS, indicating that higher NC values were consistently associated with lower muscle strength relative to body mass.

**TABLE 3:** Correlation coefficients of neck circumference with anthropometric and cardiovascular risk factors (*n* = 64).

Variables	Correlation coefficient		
	*R*	IC 95%	*p* value
Body mass (kg)	0.72^a^	[0.58–0.82]	< 0.05^∗^
BMI (kg/m^2^)	0.69^a^	[0.52–0.79]	< 0.05^∗^
Waist circumference (cm)	0.66^a^	[0.47–0.77]	< 0.05^∗^
∑ Skinfolds (mm)	0.45^a^	[0.23–0.63]	< 0.05^∗^
SBP (mmHg)	0.37^a^	[0.13–0.56]	< 0.05^∗^
DPB (mmHg)	0.36^a^	[0.12–0.56]	< 0.05^∗^
Glycated hemoglobin (%)	0.25^b^	[−0.02–0.46]	< 0.05^∗^
Fasting glucose (mg/dL)	0.23^b^	[−0.01–0.45]	0.06
TG/HDL	0.23^b^	[−0.02–0.48]	0.06
Index TG/glucose	0.22^a^	[0.00–0.44]	0.08
Triglycerides (mg/dL)	0.22^b^	[−0.03–0.41]	0.08
CRP (mg/L)	0.15^b^	[−0.10–0.37]	0.24
Relative muscle strength	−0.43^b^	[−0.60–−0.21]	< 0.05^∗^

*Note:* Legend: ∑ Skinfolds = sum of skinfolds (triceps, subscapular, abdomen, and thigh); TG/HDL = triglycerides/high‐density lipoprotein. Non‐normal variables, 95% CIs estimated by bias‐corrected accelerated (BCa) bootstrapping, 1000 samples.

Abbreviations: BMI, body mass index; CRP, C‐reactive protein; DBP, diastolic blood pressure; SBP, systolic blood pressure.

^a^Pearson.

^b^Spearman.

^∗^ *p* < 0.05 = statistically significant.

## 4. Discussion

The main findings of our study were as follows: (a) The group with higher NC (≥ 33.5 cm) showed greater body weight, BMI, waist circumference, DBP, and sum of skinfolds (indicating higher adiposity); (b) RMS was lower in elderly women with higher NC values, with effect sizes suggesting a meaningful difference between groups; baseline characteristics indicated a higher prevalence of cardiometabolic conditions among participants with higher NC, suggesting a potential role of NC as a marker of cardiometabolic risk. However, no significant differences were observed between groups in the other physical and functional tests (sit to stand, biceps curl, TUG, absolute handgrip strength, and 6MWT); and (c) an association was observed between NC and cardiovascular risk indicators, including body mass, BMI, waist circumference, sum of skinfolds, and systolic and diastolic blood pressure.

The anthropometric, biochemical, and functional indicators used in this study are internationally recognized and commonly applied in elderly populations as reliable markers of general and cardiovascular health [2], including BMI, blood pressure, fasting glucose, glycated hemoglobin, lipid profile, CRP, waist circumference, and the sum of skinfolds. Furthermore, the functional tests employed are well‐validated in the literature [12] and demonstrated excellent reliability in our study, with ICC exceeding 0.7.

Our findings indicate that NC is positively associated with visceral adiposity, BMI, subcutaneous fat (as indicated by the sum of skinfolds), and blood pressure, supporting its potential as a proxy for cardiovascular risk. These findings are consistent with previous studies suggesting that NC can predict insulin resistance [10], adiposity, and visceral fat [18] and serve as an important marker of cardiovascular risk [4, 5]. Moreover, NC offers practical advantages, including low cost, ease of measurement, minimal participant burden, and applicability in clinical and epidemiological settings.

The absence of consistent associations between NC and biochemical biomarkers in our study may be partially explained by the characteristics of the sample. As shown in Table 1, biomarker levels such as fasting glucose, glycated hemoglobin, and lipid‐related indicators were relatively similar between NC groups, despite marked differences in anthropometric measures and cardiovascular risk profiles. In addition, a high prevalence of pharmacological treatment for chronic conditions, including diabetes, dyslipidemia, and hypertension, was observed across both groups (Supporting Table 2), which may have attenuated between‐group differences in circulating biomarkers.

To the best of our knowledge, no previous study has investigated the association between NC and functional tests in older women. Our initial hypothesis was that individuals with higher adiposity and BMI would experience greater difficulty performing daily tasks. However, no significant association was found between NC and functional performance in this population. This finding may reflect limited variability in physical function among participants, possibly due to a relatively homogeneous functional profile and preserved mobility status.

Differences in RMS were not mirrored by performance‐based functional tests. The absence of differences in functional performance may be explained by several factors. Relative strength derived from handgrip testing is a more sensitive marker of neuromuscular decline, whereas functional tests tend to detect limitations only at more advanced stages [12]. Tests such as the sit to stand, TUG, and 6MWT may present ceiling effects in independently living older adults with preserved mobility, which could reduce their ability to discriminate subtle functional differences between groups with relatively high functional capacity, as observed in the present sample. These factors may explain why relative strength differed between groups, with effect sizes suggesting meaningful differences, whereas functional performance did not.

Strength is an earlier, more proximal marker of functional decline (dynapenia): Low midlife handgrip predicts disability decades later [19], and the European consensus on sarcopenia [20] prioritizes low strength for prognosis purposes. Power‐oriented sit‐to‐stand metrics may capture earlier changes, supporting the view that declines in strength can precede measurable functional impairment. Consistent with this mechanism, women with higher NC in our sample exhibited lower relative strength, likely because they have greater total adiposity; as relative strength is normalized to body mass, an increase in the denominator without a proportional gain in force yields a lower value [4].

From a clinical perspective, the combination of reduced RMS and preserved functional performance may represent an early stage of functional decline and neuromuscular impairment, consistent with the concept of dynapenia [21]. In this context, elevated NC coupled with lower RMS, despite normal functional test performance, may indicate that neuromuscular impairments are already present but not yet detectable by conventional performance‐based tests such as the TUG or 6MWT.

Older women presenting this profile may benefit from early preventive interventions, including resistance training programs aimed at preserving neuromuscular function, as well as nutritional strategies emphasizing adequate protein intake [20]. Therefore, RMS may serve not only as a biomarker of current functional status but also as a sensitive indicator of early functional vulnerability, potentially helping to highlight individuals who may be at increased risk of future functional decline [20, 21].

Although no significant associations were observed between NC and functional performance, it is possible that the study lacked sufficient statistical power to detect small‐to‐moderate effects for these outcomes. Given the relatively preserved functional status of the sample and the low variability in TUG and 6MWT scores, ceiling effects may have further limited statistical power.

Previous studies have identified muscle strength as a predictor of cardiovascular risk [13] and mortality [22, 23]. Therefore, it appears reasonable to consider NC as a potential anthropometric correlate of cardiovascular risk in older women, given its association with blood pressure, BMI, adiposity, and RMS [24]. A study [25] investigated the possible association of NC with cardiometabolic risk factors and found the possible cutoff points of NC for the diagnosis of metabolic syndrome. The results demonstrated that NC was significantly associated with central obesity, hypertension, hypertriglyceridemia, impaired fasting glucose, low HDL cholesterol, and the presence of metabolic syndrome.

Although a cutoff point of 33.5 cm was adopted based on previous evidence, it is important to emphasize that NC demonstrated consistent associations with cardiometabolic risk factors and RMS when analyzed as a continuous variable. This reinforces the clinical value of NC as a simple and scalable anthropometric marker capable of capturing gradations of risk. Although categorical analyses facilitate clinical interpretation and risk stratification, continuous analyses preserve statistical power and avoid information loss associated with dichotomization. Therefore, the consistency of associations observed using both analytical approaches strengthens the robustness of the findings.

Given the evaluation of multiple endpoints and the exploratory nature of this study, the findings should be interpreted with caution. Emphasis should be placed on the magnitude, direction, and consistency of the observed associations rather than on isolated statistical significance. This approach allows a more comprehensive interpretation of the data, particularly in studies with relatively small sample sizes.

The study has some limitations: (a) the relatively small sample size limited the number of covariates that could be included in multivariate analyses, increasing the risk of overfitting; thus, residual confounding from factors such as smoking, alcohol intake, socioeconomic status, and medication classes, doses, or treatment duration cannot be ruled out, as these variables were not adjusted for and may have influenced cardiometabolic parameters; (b) the sample consisted exclusively of older women from a single city, which restricts external validity; and (c) participants were older women with preserved functional capacity, which may have contributed to ceiling effects in functional tests and limits the generalizability of the findings to populations with greater functional impairment. Additionally, the relatively small sample size may have limited the statistical power to detect small differences in functional performance outcomes. The sample size was determined based on the primary outcome of RMS.

Despite these limitations, the study contributes to a better understanding of NC as a marker in older women, and future research should employ longitudinal designs with larger, more diverse populations, including men, to strengthen causal inference and generalizability.

## 5. Conclusion

The present study showed that older women with higher NC values (≥ 33.5 cm) had greater body weight, BMI, waist circumference, blood pressure, and sum of skinfolds. Additionally, RMS was lower in women with higher NC values, whereas no consistent differences were observed between NC and functional performance. NC may be a simple and practical anthropometric indicator associated with cardiovascular risk in older women, but further studies with larger, more diverse samples and longitudinal studies are required to confirm these findings.

## Funding

This research did not receive any specific grant from funding agencies in the public, commercial, or not‐for‐profit sectors. This study was funded by the authors.

## Disclosure

The funders had no role in the study design, collection, analyses, or interpretation of data, in the writing of the manuscript, or in the decision to publish the results.

All authors have read and approved the submission of the manuscript; the manuscript has not been published and is not being considered for publication elsewhere, in whole or in part, in any language, except as an abstract.

The work reflected in this manuscript is original and unpublished anywhere. It is not under editorial consideration anywhere else.

## Ethics Statement

The present study has been approved by the institutional ethics committee of the local university (CAAE 30059120.3.0000.5701) and complied with the ethical standards laid down in the 1964 Declaration of Helsinki and its later amendments. All participants gave their informed consent before inclusion in the study. Details that might disclose the identity of the participants under study were omitted.

## Conflicts of Interest

The authors declare no conflicts of interest.

## Supporting Information

Additional supporting information can be found online in the Supporting Information section.

## Supporting information


**Supporting Information 1** Supporting Table S1 presents the STROBE checklist.

**Supporting Information 2** Supporting Table S2 summarizes the prevalence of comorbidities and medication use according to neck circumference groups (< 33.5 and ≥ 33.5 cm).

## Data Availability

The data supporting the findings of this study are available from the corresponding author upon reasonable request.
