# Supplementary material for: Association Between Neck Circumference, Cardiovascular Risk Factors, and Relative Muscle Strength in Older Women
Source: J Aging Res. 2026 Apr 10;2026:2875747. doi: 10.1155/jare/2875747 (PMC13069176; doi:10.1155/jare/2875747)
Supplement: Supplementary file 2 — Supporting Information 2 Supporting Table S2 summarizes the prevalence of comorbidities and medication use according to neck circumference groups (< 33.5 and ≥ 33.5 cm). [file JARE-2026-2875747-s002.docx]

| **Variable** | **<33.5 cm (n=33)** | **≥33.5 cm (n=31)** | **p-value** |
| --- | --- | --- | --- |
| **Comorbidities** |  |  |  |
| Diabetes | 10 (30.3%) | 17 (54.8%) | <0.05 |
| Dyslipidemia | 14 (42.4%) | 21 (67.7%) | <0.05 |
| Hypertension | 17 (51.5%) | 22 (71.0%) | 0.11 |
| **Antihypertensive drugs** |  |  |  |
| ARBs | 16 (48.5%) | 19 (63.3%) | 0.23 |
| Diuretics | 6 (18.2%) | 4 (13.3%) | 0.73 |
| ACE inhibitors | 3 (9.1%) | 9 (30%) | 0.05 |
| Calcium channel blockers | 2 (6.1%) | 7 (23.3%) | 0.07 |
| **Lipid-lowering drugs** |  |  |  |
| Statins | 10 (30.3%) | 13 (43.3%) | 0.28 |
| Fibrates | 0 (0%) | 4 (13.3%) | 0.04 |
| **Antidiabetic drugs** |  |  |  |
| Insulin | 1 (3%) | 1 (3.3%) | 1.00 |
| Sulfonylureas | 3 (9.1%) | 1 (3.3%) | 0.61 |
| Biguanide | 9 (27.3%) | 13 (43.3%) | 0.18 |

**Supplementary Table 2.** Baseline characteristics according to neck circumference groups

**Legend**: Values are presented as absolute frequency and percentage [n (%)]. Comparisons between neck circumference groups (<33.5 cm vs ≥33.5 cm) were performed using the chi-square test or Fisher’s exact test when appropriate. ARBs = angiotensin II receptor blockers; ACE inhibitors = angiotensin-converting enzyme inhibitors.
